# Síndrome de feocromocitoma-paraganglioma de tipo 5 como causa de hipertensión arterial en una paciente colombiana: reporte de caso

**DOI:** 10.7705/biomedica.7152

**Published:** 2024-05-31

**Authors:** Juan Morales, Daniela Arturo, Miguel Folleco

**Affiliations:** 1 Departamento de Medicina Interna, Hospital Universitario del Valle, Universidad del Valle, Cali, Colombia Universidad del Valle Hospital Universitario del Valle Universidad del Valle Cali Colombia; 2 Escuela de Ciencias Básicas, Grupo de Investigación en Enfermedades Congénitas del Metabolismo, Universidad del Valle, Cali, Colombia Universidad del Valle Escuela de Ciencias Básicas Grupo de Investigación en Enfermedades Congénitas del Metabolismo Universidad del Valle Cali Colombia; 3 Sección de Endocrinología, Clínica Imbanaco, Cali, Colombia Clínica Imbanaco Sección de Endocrinología Clínica Imbanaco Cali Colombia

**Keywords:** feocromocitoma, paraganglioma, succinato deshidrogenasa, hipertensión, tumores neuroendocrinos, imagen multimodal, medicina de precisión, Pheochromocytoma, paraganglioma, succinate dehydrogenase, hypertension, neuroendocrine tumors, multimodal imaging, precision medicine

## Abstract

El feocromocitoma es un tumor derivado de las células de la cresta neural con la capacidad de producir sustancias simpaticomiméticas y, por ende, un cuadro clínico particular. Causa menos del 1 % de los casos de hipertensión arterial sistémica y su incidencia se estima entre 0,4 y 0,6 casos por 100.000 personas cada año, con una supervivencia media de siete años. De todos los tumores sólidos, el feocromocitoma tiene un mayor componente genético, que puede heredarse hasta en el 40 % de los casos. Una vez diagnosticada la enfermedad, se debe definir el tratamiento y el pronóstico, en parte condicionados por las variantes genéticas asociadas, en especial *RET*, *SDHx*, *VHL* y *NF1*.

Se presenta el caso de una mujer joven con dolor abdominal e hipertensión arterial sistémica, a quien se le diagnosticó feocromocitoma. Al secuenciar el exoma, se identificó una variante patogénica extremadamente rara y de reciente descubrimiento: *SDHA*: c.1A>C (p.Met1Leu). La paciente respondió adecuadamente al tratamiento quirúrgico y continuó en seguimiento sin recurrencias.

El abordaje diagnóstico de los pacientes con feocromocitoma comienza con la sospecha clínica, seguida de la medición de determinados metabolitos en sangre y orina, y, finalmente, los estudios de imagenología. Los desarrollos tecnológicos actuales permiten la aplicación de la medicina de precisión en este campo. En este caso de feocromocitoma, se identificó un componente genético importante que no solo afecta al paciente, sino también, a sus familiares. La tamización adecuada del caso índice permite identificar mutaciones y caracterizar mejor la enfermedad.

## Abstract (en)

Pheochromocytoma is a tumor derived from neural crest cells able to produce sympathomimetic substances and, hence, a particular clinical picture. It is responsible for less than 1% of high blood pressure cases, with an estimated incidence between 0.4 and 0.6 cases per 100,000 people each year, and an average survival of seven years. Pheochromocytoma is a solid tumor with a high genetic component, as heritability can reach 40%. Once diagnosed, its treatment and prognosis are partly conditioned by the associated pathogenic variants that can be documented, especially those related to *RET*, *SDHx*, *VHL*, and *NF1* genes.

We present the case of a young woman with abdominal pain and high blood pressure, who was found to have a pheochromocytoma. Genetic testing detected a rare and recently discovered pathogenic variant: the *SDHA*:c.1A>C (p.Met1Leu). The patient responded adequately to the surgical treatment and continued the follow-up without documented recurrences.

The diagnostic approach for pheochromocytoma patients must start with a clinical suspicion, followed by metabolite measurement in blood and urine, and finally, imaging. Currently, technology development allows precision medicine applicability. In this case of pheochromocytoma, recent developments in precision medicine resulted in the detection of associated genetic components involving the patient and her family. Adequate screening of the index patient is required for documenting pathogenic variants and better characterizing the disease.

Los feocromocitomas y paragangliomas son tumores neuroendocrinos ricamente vascularizados, derivados de las células cromafines provenientes de la cresta neural ^1^. Su principal diferencia radica en su localización: los primeros se presentan en las glándulas suprarrenales, mientras que los últimos se presentan en otros sitios diferentes. Su incidencia en la población general es baja, entre 0,4 y 0,6 casos por 100,000 personas por año ^2^^,^^3^. La edad usual de presentación es entre los 30 y los 50 años, sin encontrarse diferencias entre hombres y mujeres. En Colombia, los datos son muy escasos. Además de los reportes de caso, se han descrito dos series: la primera en 1980 con seis pacientes y la segunda, en el 2016, en la que se documentaron 11 casos en una ventana de 10 años. Sin embargo, se desconocen los datos a nivel poblacional ^4^.

La primera descripción clínica completa del feocromocitoma fue realizada por Felix Fränkel en 1884. Se trató de una mujer de 18 años, llamada Minna Roll, quien presentaba ataques de pocos minutos de duración de palpitaciones, cefalea, ansiedad y vértigo. Los episodios se hicieron más frecuentes y se acompañaron de fiebre, diaforesis, visión borrosa, hiporexia y mialgias. Posteriormente, la paciente presentó alteración del estado de conciencia y dolor torácico, y sufrió una muerte súbita ^5^.

La descripción histopatológica fue realizada por Max Schottelius. Actualmente, continúa determinándose el perfil de la enfermedad, de acuerdo con las características clínicas, bioquímicas y genéticas ^3^.

La principal característica bioquímica de estos tumores es la producción y secreción de catecolaminas (adrenalina, noradrenalina y dopamina) o metanefrinas (metanefrina y normetanefrina) en grandes cantidades y con la pérdida de la autorregulación. Debido a esto, se produce una amplia variedad de síntomas y signos característicos de la enfermedad, como arritmias, temblores, palpitaciones, sudoración y cefalea. Las tres últimas son conocidas como la triada clásica del feocromocitoma. Por la poca especificidad de los signos y síntomas, se han descrito, por lo menos, 30 enfermedades médicas que pueden simular el cuadro clínico del feocromocitoma ^3^.

Entre los tumores sólidos, se estima que los feocromocitomas tienen la mayor predisposición genética, pues hasta en el 40 % de los casos pueden encontrarse genes patógenos, en ocasiones, asociados con síndromes clínicos como el de von Hippel-Lindau, la neurofibromatosis de tipo 1 y el síndrome de feocromocitoma-paraganglioma ^1^.

Gracias al desarrollo de las pruebas moleculares, la descripción y correlación entre el genotipo y el fenotipo de las diferentes variantes es un tema activo de investigación. Sin embargo la evolución y el pronóstico de los pacientes portadores aún no son claros ^6^^,^^7^.

## Presentación del caso

Se presenta el caso de una mujer de 35 años, con un cuadro clínico de dos años de evolución consistente en dolor abdominal de intensidad moderada, episódico, de distribución difusa y sin factores desencadenantes. A pesar, de consultar en múltiples ocasiones al servicio médico y recibir manejo analgésico, la sintomatología persistía. En la revisión por sistemas, no se describieron síntomas adicionales.

Al indagar sobre sus antecedentes, la paciente comentó padecer hipertensión arterial tratada con tres antihipertensivos diferentes, tuvo tres embarazos con partos sin complicaciones y el antecedente familiar de una hermana con una masa abdominal, que había fallecido sin una causa clara.

En el el examen físico, se encontró una presión arterial de 149/94 mm Hg y una frecuencia cardíaca de 102 latidos por minuto. En la exploración cardiopulmonar y abdominal no se identificaron hallazgos significativos. Con base en lo anterior, se consideró a la paciente como una mujer joven con hipertensión arterial secundaria, en quien debían estudiarse las posibles causas de su enfermedad.

Los resultados de los laboratorios mostraron: anemia leve, con un volumen corpuscular medio disminuido, ferritina superior a los 2.000 ng/ ml, función renal sin alteraciones, leve hipocalcemia y bioquímica hepática dentro de los rangos de normalidad. Los niveles de la hormona estimulante de la tiroides eran normales, tenía una hemoglobina glicosilada de 6,4 %, y su electrocardiograma de superficie evidenció trastornos inespecíficos de la repolarización.

**Figura 1:**
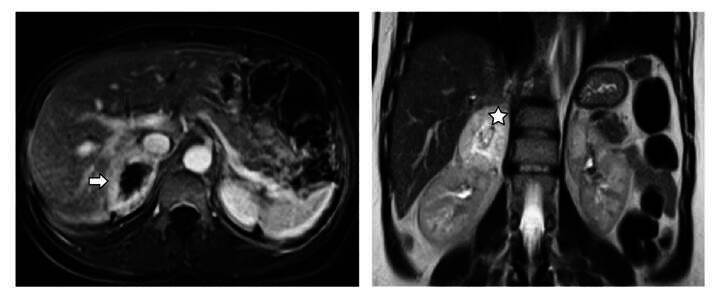
Estudio inicial de resonancia magnética con contraste de abdomen. Izquierda: secuencia T1; cortes axiales en los que se observa una lesión tumoral suprarrenal derecha con centro necrótico y atenuación de la intensidad de la señal (flecha). Hay infiltración de la vena cava inferior retrohepática con un trombo en su interior. Derecha: secuencia T2; cortes coronales; hacia el polo superior renal derecho, se puede apreciar una masa tumoral con realce que genera compresión hepática y renal (estrella).

Con base en estos resultados y el antecedente de dolor abdominal de etiología inespecífica, se procedió a realizar estudios de imagenología. El ecocardiograma transtorácico solo mostró remodelado concéntrico del ventrículo izquierdo, con una fracción de eyección del 75 %. En la tomografía contrastada de abdomen se localizó, a la altura de la glándula suprarrenal derecha, una masa de 6,4 x 5 x 6 cm, sin calcificaciones, con una densidad de 29 UH, cuya densidad aumentaba con la administración del medio de contraste -sobre todo en la porción periférica- y que, además, generaba un efecto compresivo sobre la vena cava inferior. Se complementó este estudio con una resonancia por fuera de las glándulas suprarrenales (figura 1).

De acuerdo con la imagen anterior y los datos clínicos de la paciente, se planteó un feocromocitoma como un diagnóstico muy probable. Para comprobarlo, se solicitaron valores de metanefrinas en plasma, cuyos resultados fueron anormales: metanefrinas, 85,6 pg/ml (valor de referencia < 65 pg/ml), y normetanefrinas, 4.539 pg/ml (valor de referencia < 196 pg/ml).

Dado el contexto clínico, los resultados de las imágenes diagnósticas y las pruebas de laboratorio, se estableció un diagnóstico definitivo de feocromocitoma.

La paciente fue informada sobre su enfermedad y las opciones terapéuticas, y optó por el manejo quirúrgico. Previo al bloqueo adrenérgico secuencial con α y β-bloqueadores, se extirpó la masa por vía abierta, sin complicaciones perioperatorias.

En el informe de histopatología, se reportó un carcinoma neuroendocrino bien diferenciado e infiltrante de 9 x 5 x 6 cm, compromiso de la cápsula de resección e infiltración de la pared de la vena cava inferior. El informe complementario de la inmunohistoquímica fue positivo para la proteína de las células de Schwann S100 y cromogranina A, hallazgos típicos de los tumores neuroendocrinos ^8^, además de un índice de proliferación de la Ki-67 de 2,3 %.

Tras recibir el alta hospitalaria, la paciente fue sometida a un estudio molecular mediante secuenciación del exoma completo, utilizando la plataforma MGIEasy Exome Capture V5. No se identificaron mutaciones en el gen *RET*, pero se detectó una variante patógena registrada en la base de datos ClinVar como *Variation ID* 8744 para variantes PS1, según las recomendaciones del *American College of Medical Genetics* (ACMG).

La variante consiste en una transición de adenina por citosina (A>C) en la posición 218.356 del cromosoma 5, lo que genera un cambio del aminoácido metionina por leucina en la primera posición de la proteína (p.Met1Leu) codificada por el gen de la subunidad A de la enzima succinato deshidrogenasa (*SDHA*) en condición heterocigota. Esta variante genera una pérdida del sitio -codón- de inicio en el marco de lectura del transcrito, lo que origina una proteína deletérea. Esta variante se ha asociado con feocromocitoma-paraganglioma de tipo hereditario (cuadro 1).

**Cuadro 1:** Variante patogénica reportada mediante secuenciación del exoma

Gen	Cambio de nucleótido	Cambio de aminoácido	Cigosidad
*SDHA*	c.1A>C	p.Met1Leu	Heterocigoto

*SDHA*: succinato deshidrogenasa subunidad A

**Figura 2:**
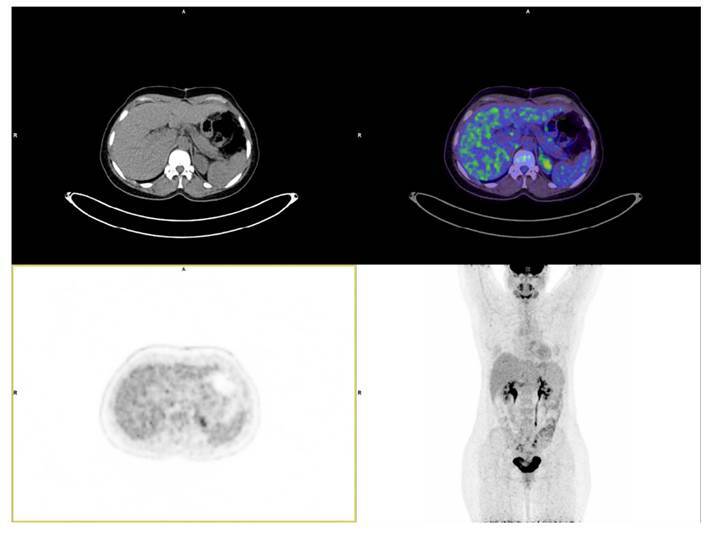
Tomografía por emisión de positrones con tomografía multicorte (PET/CT) tomada un año después de la resección tumoral. El trazador utilizado fue ^18^F-FDG. El estudio fue negativo para actividad hipermetabólica anormal.

Al año de la resección quirúrgica, se practicó una tomografía por emisión de positrones con tomografía multicorte (PET/CT) con ^18^F-FDG, que no evidenció actividad hipermetabólica anormal (figura 2) y se hizo un análisis de metanefrinas en orina de 24 horas con resultado normal. Los valores de presión arterial se normalizaron, la hemoglobina glicosilada mostró tendencia a disminuir y el dolor abdominal desapareció. Finalmente, la paciente fue remitida para valoración por genética para consejería y para continuar con el proceso de tamización en sus familiares.

### Consideraciones éticas

La paciente autorizó el uso de sus datos clínicos mediante un consentimiento informado. Se garantizó la confidencialidad de su identidad en todo momento.

## Discusión

Se presenta el caso de una mujer joven con hipertensión arterial sistémica secundaria y dolor abdominal, cuya causa, aunque mencionada en la literatura, es vista rara vez en la práctica clínica debido a su muy escasa incidencia.

Se estima que hasta uno de cada tres pacientes con feocromocitoma presenta variantes en su línea germinal que pueden estar asociadas con diferentes síndromes genéticos ^3^^,^^9^, en los cuales la correlación entre el fenotipo y el genotipo es esencial (cuadro 2). En esta paciente no se encontraron variantes del gen *RET* y, dado que no evidenciaba un fenotipo sugestivo de síndrome de von Hippel-Lindau o neurofibromatosis de tipo 1, no se analizaron los genes asociados con estas enfermedades.

**Cuadro 2:** Características genéticas y fenotípicas de los síndromes asociados con feocromocitoma-paraganglioma

Gen mutado	Síndrome asociado	Tumores asociados nocromafines	Localización suprarrenal	Metástasis
*VHL*	von Hippel-Lindau	Hemangioblastoma en retina y sistema nervioso central, carcinoma renal, tumores endolinfáticos del oído interno, tumor neuroendocrino pancreático	> 50 %	Hasta el 9 %
*NF1*	Neurofibromatosis de tipo 1	Neurofibromas cutáneos, tumores de vaina de nervio periférico, cáncer de mama	> 50 %	Hasta el 9 %
*RET*	MEN-2	Carcinoma medular de tiroides, hiperparatiroidismo primario	> 50 %	< 1 %
*SDHD*	PGL-1	Adenoma pituitario, tumor del estroma gastrointestinal, carcinoma renal	Hasta el 24 %	Hasta el 9 %
*SDHAF2*	PGL-2	Desconocido	Hasta el 9 %	Desconocido
*SDHC*	PGL-3	Igual que PGL-1	Hasta el 9 %	Desconocido
*SDHB*	PGL-4	Igual que PGL-1	50 %	Hasta el 50 %
*SDHA*	PGL-5	Igual que PGL-1	50 %	Hasta el 9 %
*MAX*	No asociado	Carcinoma renal	> 50 %	Hasta el 9 %
*TMEN127*	No asociado	No asociado	> 50 %	Hasta el 24 %
*HIF2*	No asociado	Policitemia	Desconocido	Desconocido

VHL: von Hippel-Lindau, NF 1: neurofibromatosis de tipo 1; MEN 2: neoplasia endocrina múltiple de tipo 2, SDH: succinato deshidrogenasa, PGL: síndrome de feocromocitoma-paraganglioma

Entre los genes relacionados con el feocromocitoma, se encuentra el complejo enzimático de la succinato deshidrogenasa (SDHx), clave en el ciclo de Krebs y con un rol como supresor de tumores ^10^^,^^11^. En la paciente, se reportó una variante patógena del gen *SDHA*, la cual se presenta, aproximadamente, en el 3 % de todos los feocromocitomas. La descripción de esta variante y su relación con el feocromocitoma se inició hace pocos años, de manera que los casos descritos en todo el mundo son escasos y apenas se está dilucidando la relación entre fenotipo y genotipo ^7^.

El gen *SDHA* codifica una subunidad catalítica importante de la succinato-ubicuinona oxidorreductasa (EC 1.3.5.1), el complejo II de la cadena respiratoria mitocondrial, también conocido como succinato deshidrogenasa. Este complejo consta de cuatro unidades polipeptídicas codificadas en el núcleo que, en orden de masa molecular decreciente, son la subunidad de flavoproteína (SDHA), la subunidad de hierro y azufre (SDHB; 185470) y las subunidades de proteínas integrales de membrana de anclaje SDHC (602413) y SDHD (602690) ^12^.

Las feocromocitomas son tumores de células cromafines derivados de la cresta neural que secretan catecolaminas o metanefrinas. Casi del 80 al 85 % de los feocromocitomas se derivan de la médula suprarrenal, mientras que del 15 al 20 %, de tejido cromafín diferente al suprarrenal. Los tumores de células cromafines extrasuprarrenales son referidos como feocromocitomas extrasuprarrenales o paragangliomas.

El término “paraganglioma” también es utilizado para tumores derivados de tejido parasimpático en cabeza y cuello que, en su mayoría, no producen catecolaminas. Estos tumores presentan gran predisposición genética, con un patrón de herencia autosómico dominante ^13^. En los últimos 15 años, se han reportado variantes germinales en un gran número de genes de susceptibilidad a paraganglioma. Se estima que, aproximadamente, el 40 % de los pacientes tiene una variante germinal causal. También, hay varios genes que se han asociado con el feocromocitoma cuando no se presenta como parte de un síndrome (cuadro 2).

La presentación clínica inicial de la paciente es llamativa por la combinación de dolor abdominal e hipertensión arterial sistémica. En la serie de casos descrita por Jha *et al*., 5 de 10 pacientes presentaban dolor abdominal como síntoma inicial y 6 de 10 eran hipertensos ^10^.

Para el diagnóstico de feocromocitoma, se requiere la demostración bioquímica del exceso de catecolaminas o metanefrinas, acompañada de la imagen diagnóstica. En las primeras descripciones de feocromocitoma relacionado con *SDHA*, se planteó una mayor producción de metanefrina, en vez de normetanefrina. Sin embargo, según información más reciente, el exceso de normetanefrinas en estos pacientes no es inusual e, incluso, pueden presentar niveles elevados de dopamina, aunque la sobreproducción de adrenalina es poco común ^9^.

En este caso, la paciente presentaba niveles de normetanefrinas en plasma diez veces por encima del límite superior normal. De acuerdo con la evidencia actual, se considera infrecuente que los feocromocitomas asociados con *SDHA* sean no productores ^14^ y, de ser así, se debe sospechar un paraganglioma de origen parasimpático, dependiendo de su ubicación ^15^^,^^16^.

Otro aspecto para tener en cuenta en los feocromocitomas y paragangliomas asociados con el complejo SDH, es el potencial de producir metástasis. Clásicamente, se ha descrito que la variante *SDHB* tiene hasta un 50 % de riesgo de presentar metástasis, ya sean abdominales o extraabdominales ^3^. Sin embargo, al parecer este riesgo no está confinado solamente a dicho gen. Existen reportes de pacientes con feocromocitomas relacionados con *SDHA* donde hasta el 66 % tenía metástasis al momento del diagnóstico o lo desarrollaron en un periodo de 8,5 años después del mismo ^10^.

En el presente caso, a la paciente se le practicaron estudios extensos de imagenología, en los cuales no se demostró metástasis al momento del diagnóstico. Sin embargo, estos pacientes deben estar en seguimiento constante pues, aunque la cirugía sea de resección completa, no se elimina el riesgo de recurrencias y metástasis a lo largo de la vida ^8^.

La recomendación actual es el seguimiento bioquímico mediante la evaluación anual de metanefrinas en plasma u orina de 24 horas ^9^. Respecto a los estudios de imagenología, no se ha establecido una periodicidad, pero parece razonable que se practiquen cada dos a tres años ^17^. Una pregunta que surge, y que es tema de debate actualmente, es el tipo de examen de imagen que se debe practicar durante el seguimiento. Se ha demostrado que para localizar la enfermedad metastásica, la gammagrafía con yodo 123 MIBG (meta-yodo-bencilguanidina) presenta una sensibilidad menor respecto a las imágenes de tomografía por emisión de positrones.

Por lo tanto, la PET/CT es el estudio de elección durante el seguimiento, con un rendimiento similar para los trazadores 68 Ga-DOTATATE y la ^18^F-FDG. Probablemente, la menor sensibilidad registrada para el yodo 123 MIBG se deba al bajo grado de diferenciación de estos tumores, lo cual limita la captación del trazador en el tejido ^10^^,^^18^. Para la detección de recurrencias localizadas, la resonancia magnética es apropiada porque evita la radiación sin comprometer la calidad de la imagen. Para los familiares portadores asintomáticos de la mutación *SDHA*, las recomendaciones son iguales ^17^.

Actualmente, la paciente permanece asintomática, los estudios de imagen posteriores no han mostrado recurrencias (figura 2) y la medición de metanefrinas ha estado dentro de los rangos normales. La tamización de sus familiares no ha sido posible dado que residen en zona rural y tienen dificultades para el acceso al servicio de salud.

En conclusión, el feocromocitoma, aunque es un tumor infrecuente, puede presentarse con síntomas como dolor abdominal e hipertensión arterial en pacientes jóvenes. El diagnóstico debe estar dirigido por la sospecha clínica, los estudios bioquímicos y una imagen diagnóstica inicial, de preferencia una tomografía contrastada de abdomen. Una vez se tenga certeza del diagnóstico, se debe evaluar el riesgo del paciente y definir el tratamiento. En conjunto con lo anterior, se requieren estudios moleculares para identificar las variantes asociadas en estos pacientes, que no solo ayudan a establecer el pronóstico, sino también, la frecuencia del seguimiento.

La información epidemiológica de estos genes en el contexto nacional es escasa y, como ocurrió en este caso, pueden existir variantes sumamente raras o aún no descritas en las poblaciones colombianas.
